# Consumption of an omega-3 fatty acids product, INCELL AAFA™, reduced side-effects of CPT-11 (irinotecan) in mice

**DOI:** 10.1038/sj.bjc.6600175

**Published:** 2002-03-18

**Authors:** W E Hardman, M P Moyer, I L Cameron

**Affiliations:** Pennington Biomedical Research Center, Louisiana State University, 6400 Perkins Road, Baton Rouge, Louisiana, LA 70808, USA; INCELL Corporation, LLC, 12000 Network Suite B 200, San Antonio, Texas, TX 78249, USA; Department of Cellular and Structural Biology, University of Texas Health Science Center at San Antonio, 7703 Floyd Curl Drive, San Antonio, Texas, TX 78229-3900, USA

**Keywords:** omega-3 fatty acids, CPT-11, cancer chemotherapy

## Abstract

INCELL AAFA™, an omega-3 polyunsaturated fatty acid product containing a high concentration of long chain fatty acids, was tested for its ability to ameliorate the harmful side effects of CPT-11 chemotherapy including: leukopenia, anaemia, asthenia, weight loss and liver involvement. Four groups of mice were fed an AIN-76 diet modified to contain: 10% w/w corn oil (CO), 0% AAFA™; 9% CO, 1% AAFA™; 8% CO, 2% AAFA™; or 7% CO, 3% AAFA™. After 2 weeks on the diets, half of the mice received CPT-11 chemotherapy (60 mg kg^−1^ q 4 days, i.v.) the rest of the mice received vehicle for 2 weeks. It was found that 2% AAFA™ in the diet of the CPT-11 treated mice: decreased apoptotic figures in the duodenal crypts; markedly suppressed the inflammatory eicosanoid, prostaglandin E_2_ in the liver; prevented liver hypertrophy; improved white blood cell counts; significantly increased red blood cell counts; decreased numbers of CPT-11 induced immature red blood cell and micronuclei in red blood cells of the peripheral blood; increased eicosapentaenoic acid and docosahexaenoic acid in liver cell membranes and maintained normal grooming behaviour. Thus 2% AAFA™ in the diet reduced the side effects of CPT-11 treatment in mice.

*British Journal of Cancer* (2002) **86**, 983–988. DOI: 10.1038/sj/bjc/6600175 www.bjcancer.com

© 2002 Cancer Research UK

## 

We have reported that supplementing the diet of mice with fish oil increased the efficacy of edelfosine against MDA-MB 231 human breast cancer (Hardman *et al*, 1997), doxorubicin against A549 human lung cancer (Hardman *et al*, 2000) and CPT-11 against MCF-7 human breast cancer (Hardman *et al*, 1999a). Other investigators have found that supplementing the diet with omega-3 fatty acids increased the efficacy of: epirubicin against rat mammary tumours (Germain *et al*, 1999) and cyclophosphamide (Shao *et al*, 1997) or mitomycin (Shao *et al*, 1995) against MX-1 human mammary tumours. The results of *in* *vitro* studies have shown that a small amount of either eicosapentaenoic acid (EPA) or of docosahexaenoic acid (DHA), the major long chain polyunsaturated fatty acids (PUFAs) found in fish oil, added to cell culture media can cause tumour cell death but not kill cultured normal cells (de Vries and Van Noorden, 1992). Other reported benefits of omega-3 PUFA dietary supplements given prior to or during cancer therapy include: reversing tumour cell drug resistance (Das *et al*, 1998), reducing the gastrointestinal, haematological or cardiac side effects of various chemotherapeutic treatments (Hardman *et al*, 1999a; Germain *et al*, 1999; Shao *et al*, 1997), decreasing cancer cachexia (Karmali, 1996; Tisdale, 1993; Barber *et al*, 1999), and protection from alopecia (Takahata *et al*, 1999). Thus supplementing the diet with omega-3 fatty acids may be beneficial during cancer chemotherapy.

However, in past studies, the amount of fish oil used in the mouse diets (usually 10 to 20% w/w of the diet though 3% w/w was used in one study (Hardman *et al*, 1999a)) was higher than humans can easily consume. In an effort to reduce the volume of omega-3 fatty acid supplement required for benefit during cancer chemotherapy, we have recently acquired INCELL AAFA™, an omega-3 product containing a total of 55% eicosapentaenoic acid (EPA) + docosahexanoic acid (DHA). We calculated that between 2 and 3% w/w AAFA™ in the diet would contain the same amount of EPA + DHA as in a diet containing 6% fish oil thus a smaller volume of AAFA™ than of fish oil would be needed to obtain the beneficial effects of an omega-3 supplement.

We have previously reported that 3 or 6% w/w fish oil in the diet increased the efficacy of the CPT-11 against the tumour and suppressed some of the side effects of CPT-11 treatment (Hardman *et al*, 1999a). The topoisomerase inhibitor, CPT-11 has been a very effective chemotherapeutic drug, however, its use in patients is limited due to the severe side effects, especially to the intestines and to the bone marrow (Rothenberg, 1998; Rivory, 1996). Prior to performing an expensive tumour efficacy study, we wanted to determine whether a very small amount of AAFA™ (1 to 3% w/w of the diet) would lessen the side effects of CPT-11. Since this is a new product, the specific aims of this study were: (1) to determine if significant toxicity was associated with consumption of AAFA™ at levels of 1, 2 or 3% of the diet in Swiss mice; and (2) to conduct a detailed study of the side effects of CPT-11 if 0, 1, 2 or 3% INCELL AAFA™ was incorporated in a diet containing a total of 10% fat.

## MATERIALS AND METHODS

### Diet preparation

Dry ingredients were measured and mixed well in bulk, then oil was thoroughly blended in at 10% of the weight of an aliquot of dry ingredients. The final compositions of the diets were (g per 100 g without water): total oil, 10; sugar, 47.4; casein, 18.9; cornstarch, 14.2; AIN-76 vitamin mix, 0.97; AIN-76 mineral mix, 3.38; choline bitartrate, 0.18; DL-methionine, 0.27; cellulose, 4.7. Water was added as needed to hold ingredients together, food was compressed into a flat pan and cut into daily portions for each cage. Food was stored in air tight containers at −20°C until fed to the mice. Mice were fed fresh food and old food was removed from the cage every afternoon, 7 days per week.

### Drug preparation

CPT-11 was obtained as irinotecan hydrochloride (Pharmacia & Upjohn, Kalamazoo, MI, USA). It was prepared, according to the manufacturer's directions, to duplicate the clinical formulation (Camptosar™). The prepared CPT-11 contained: 20 mg ml^−1^ irinotecan hydrochloride, 45 mg ml^−1^ sorbitol (Sigma, St. Louis, MO, USA) and 0.9 mg ml^−1^ lactic acid (Sigma, St. Louis, MO, USA) and was pH adjusted to 3–3.8. The solution was warmed in a 100°C water bath to dissolve the CPT-11. A dose of 60 mg CPT-11 per kg body weight (about 0.08 ml per 28 g mouse) was injected into the lateral tail vein of each treated mouse, once every 4 days for 2 weeks. We have shown this dose to effectively suppress the growth of lung, colon and breast cancer xenografts (Hardman *et al*, 1999b) and that the efficacy of this dose was increased by supplementing the diet of xenograft bearing mice with fish oil (Hardman *et al*, 1999a).

### Experimental design

The experimental protocol was approved by the The University of Texas Health Science Center at San Antonio Institutional Animal Care and Use Committee. The UTHSCSA, as an institution conducting research, teaching, and education using laboratory animals, adheres to United States Federal regulations as published in the Animal Welfare Act (AWA); the Guide for the Care and Use of Laboratory Animals (Guide); the Public Health Service Policy; and the US Government Principles Regarding the Care and Use of Animals. This research meets all guidelines of the United Kingdom Co-ordinating Committee on Cancer Research guidelines for the welfare of animals in experimental neoplasia (Workman *et al*, 1998).

Forty male Swiss mice, 6 weeks old, were obtained and ears were marked for unique identification. Mice were housed five per cage in UTHSCSA Laboratory Animal Resources facilities. After 24 h acclimatisation, mice were weighed and the food was changed to one of four defined diets. The AIN-76 based, 10% total fat diets are defined above. Ten mice were designated to consume each diet. All mice were weighed three times weekly throughout the experiment.

Mice consumed the diet for 2 weeks prior to initiation of chemotherapy with CPT-11. Treatment with CPT-11 was initiated for half of the mice (one cage of five mice) on each diet and was administered at 60 mg kg^−1^ body weight, i.v. in a lateral tail vein, once every fourth day for 2 weeks. Control mice received an equivalent volume of the CPT-11 vehicle.

Mice were killed 24 h after the last CPT-11 treatment. At necropsy, mice were weighed, anaesthesised and blood was drawn via cardiac puncture. The liver and duodenum were removed, weighed then part was fixed in Omnifix® and part was quick frozen in liquid nitrogen for later analyses. The fixed duodenum was oriented in cassettes then processed to paraffin blocks. Paraffin sections were cut 4 μm thick, histological slides of the duodenum were prepared and stained with H&E. Only complete midaxially sectioned crypts on H&E stained slides of duodenum were selected for analyses of crypt height and number of apoptotic figures. Apoptotic events were identified by the morphological parameters of condensation and marginalisation of the nuclear chromatin, cell shrinking, and fragmentation of the cell into apoptotic bodies, as previously reported (Hardman *et al*, 1999a).

### Blood counts

The peripheral blood specimen was placed in an EDTA containing tube and sent to Laboratory Animal Resources for complete blood counts using a Cell-Dyn 3500R blood analyser with veterinary pack.

### Micronuclei and PCE counts

Micronuclei and polychromatophilic erythrocytes (PCE) were identified on acridine orange stained smears of peripheral blood, as previously reported (Vijayalaxmi *et al*, 1997). Fields containing a single layer of erythrocytes were identified. At least eight fields containing a total of 3000 to 4000 cells were identified on each slide and the number of micronuclei and PCEs were counted and recorded.

### Gas chromatography

Gas chromatography (as previously published (Hardman *et al*, 2001)) was used to illustrate the incorporation of diet specific fatty acids into the mitochondrial and microsomal membranes of liver of mice that consumed the 0% AAFA™ and the 2% AAFA™ diet. The fatty acid methyl esters were reported as the per cent of the total methylated fatty acids (area under the curve). There were no significant differences in the lipid content of the microsomal and mitochondrial fractions of each specimen so mean membrane fatty acids are reported to illustrate that n-3 PUFAs were incorporated into the membranes of the liver of mice that consumed 2% AAFA™.

### PGE_2_ assay

The bicyclo-PGE_2_ enzyme immunoassay kit (Cayman Chemical, Ann Arbor, MI, USA) was used according to the manufacturer's instructions to assay PGE_2_ in liver homogenates. This kit measures both PGE_2_ and the metabolites of PGE_2_ in tissues and plasma. Since PGE_2_ is quickly metabolised, the bicyclo-assay provides a more reliable estimate of total PGE_2_ in samples than assay of PGE_2_ alone.

### Coat quality as a behavioural measure of well-being

Since animals cannot be questioned for how well they feel, behavioural measures can be used as surrogates. One such measure is coat quality as a reflection of normal healthy grooming behaviour. Comparative evaluation of shiny, clean, well-groomed coats *vs* scruffy, matted coats was done by daily observation with results documented by photography. Each group of mice was lined up for a picture during the time that the mice were anaesthetised for their last CPT-11 injection. The appearance of the mice was documented using a Kodak MDS-120 digital camera and Polaroid Photo Max software. Digital images were saved, converted to grayscale and the composite picture was made using PowerPoint.

### Statistical analyses

**Figure 1:**
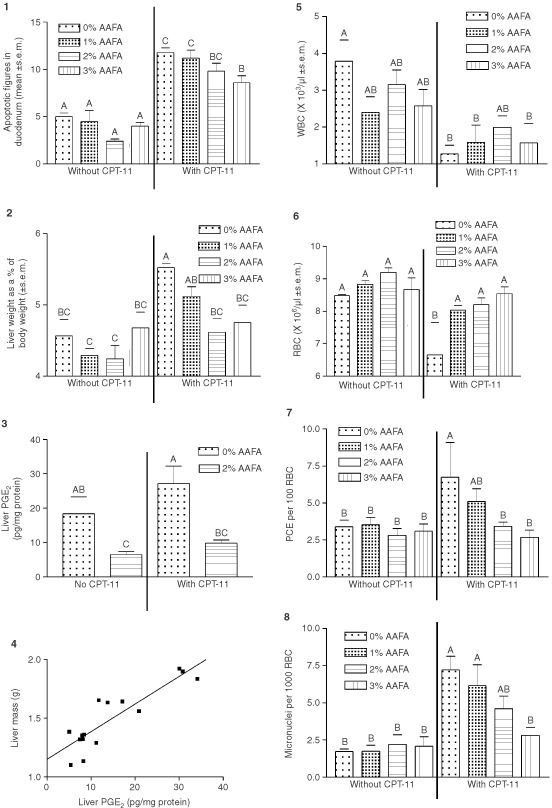
Graphs 1 to 3 and 5 to 8 illustrate the results of one-way analysis of variance of the data followed by Student–Newman–Keuls multiple range tests as appropriate. The mean±s.e.m. of five mice in each group is presented graphically, the Y-axis of each graph is labelled to identify each set of data. On graphs 1 to 3 and 5 to 8 groups that do not share a capital letter are significantly different (*P*<0.05). Graph 4 shows the linear regression analysis (*n*=15, *r*=0.882, slope is significantly different from 0, *P*<0.0001) of liver weight and liver PGE_2_ of individual mice which consumed either 0 or 2% AAFA™.

Most data was analysed by two-way analysis of variance (ANOVA) to determine main effects due to CPT-11 treatment or to AAFA™ consumption then by one-way ANOVA followed by Student-Newman-Keuls multiple range test to determine differences between individual groups. The results of two-way ANOVA tells us whether across both CPT-11 treated and untreated groups, the consumption of AAFA™ significantly altered a parameter (main effect due to AAFA™) or whether, across all diet groups, CPT-11 treatment significantly altered a parameter (main effect due to CPT-11). The results of the two-way ANOVA are presented in the text below. One-way ANOVA data is presented graphically (Figure 1

), the Y-axis of each graph is labelled to identify each set of data. The results of one-way ANOVA tells us which individual groups are significantly different. On each graph, groups which do not share a superscript are significantly different (*P*<0.05). Linear regression analyses were used to determine the correlation between liver mass and liver PGE_2_.

## RESULTS

Figure 1 is a composite of graphs showing the data. There were five mice in each group.

### Apoptotic figures and crypt height in the duodenum (graph 1)

The number of apoptotic cells found in 10 complete, midaxially sectioned duodenum crypts of each mouse was counted as an indication of intestinal damage. The results of two-way ANOVA revealed that CPT-11 treatment significantly increased the number of apoptotic cells in the duodenum crypts and that consumption of AAFA™ significantly decreased the number of apoptotic figures in the duodenum. Count of the number of cells in each crypt column revealed that there were no significant differences in number of cells in the crypt column (data not shown) due to consumption of AAFA™.

### Liver weight (graph 2)

The weight of the liver was expressed as a percentage of the body weight. Two-way ANOVA revealed that CPT-11 treatment significantly increased liver weight and that consumption of AAFA™ significantly decreased liver weight. The mean liver weights of the 2 or 3% AAFA™ fed/CPT-11 treated mice were not significantly different from the mean liver weights of the mice that were not treated with CPT-11.

### PGE_2_ level in the liver (graph 3)

Due to specimen limitations, we were only able to assay PGE_2_ in mice that consumed 0 and 2% AAFA™, with and without CPT-11 treatment. Two-way ANOVA revealed that treatment with CPT-11 increased liver PGE_2_ and that consumption of 2% AAFA™ significantly decreased liver PGE_2_.

### Correlation between liver weight and PGE_2_ content (graph 4)

Linear regression analysis illustrated the significant (*r*=0.882, *P*<0.0001) linear relationship between liver mass and liver PGE_2_.

### Blood counts (graphs 5 and 6)

Treatment with CPT-11 significantly decreased both red and white blood cell counts in mice that did not consume AAFA™. However, consumption of AAFA™ prior to and during CPT-11 treatment was associated with significantly higher red blood cell (RBC) counts and a trend (non-significant) to higher white blood cell (WBC) counts than in mice which did not consume AAFA™. The WBC and RBC counts of 2% AAFA™ fed/CPT-11 treated mice were not significantly different from those parameters in mice which did not receive CPT-11.

### Micronuclei and polychromataphilic erythrocytes (graphs 7 and 8)

Micronuclei are formed from broken strands of DNA and polychromataphilic erythrocytes (PCE) are immature erythrocytes. The results of two-way ANOVA revealed that treatment with CPT-11 significantly increased both micronuclei and PCE counts and that consumption of 2 or 3% AAFA™ prior to and during CPT-11 treatment significantly decreased micronuclei and PCE counts.

### Liver membrane fatty acids

As expected, consumption of AAFA™ increased the EPA and DHA in liver cellular membranes (expressed as a per cent of the total lipid in cellular membranes). In mice fed the 10% CO diet, the EPA and DHA fractions (mean±s.e.) were: EPA=0.08±0.06% and DHA=0.57±0.27%. In mice fed the 2% AAFA™/8% CO diet the EPA and DHA were significantly (*P*<0.01) increased to EPA=4.5±1.5% and DHA=3.92±0.30%. The fraction of arachidonic acid (range 8–10%) in cellular membranes was not significantly different due to the diet of the mice.

### Coat quality

Figure 2

**Figure 2:**
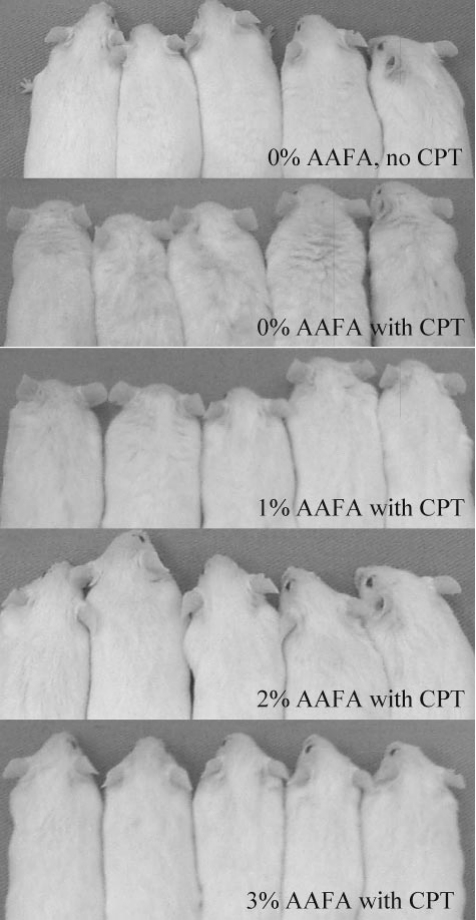
A composite of photographs of groups of mice that were treated as noted. The coats of the CPT-11 treated mice that did not consume AAFA™ look ‘scruffy’, i.e., the fur is not smooth and hair strands are stuck together, especially between the scapulae. However, mice treated with CPT-11 that consumed 1% AAFA™ look smoother and the mice that consumed 2 or 3% AAFA™ did not look different from the mice that did not receive CPT-11.

is a photographic composite of groups of mice that consumed the ‘normal’ 10% CO diet and that were not treated with CPT-11 (normal control group) and the groups of CPT-11 treated mice that consumed each of the diets. The coats of the mice that were treated with CPT-11 but that did not consume AAFA™ look ‘scruffy’, i.e., the fur was not smooth and strands of hair were stuck together, especially in the area between the scapulae. However, the coats of mice treated with CPT-11 that consumed 1% AAFA™ look smoother and the coats of mice that consumed 2 or 3% AAFA™ did not look different from the mice that did not receive CPT-11.

### Body weight

There were no significant differences in the mean body weights of the groups of mice at the beginning of CPT-11 treatment or at the time of necropsy (data not shown).

## DISCUSSION

In humans, late onset diarrhea (>24 h post CPT-11 treatment) associated with intestinal damage and decreased white blood cell counts, especially decreased neutrophil counts, are the main dose limiting side effects of treatment with CPT-11 (Rivory, 1996; Rothenberg *et al*, 1996, 1999; Rothenberg, 1998). Although the mouse model used did not develop diarrhea from the CPT-11 treatment, mice have demonstrated malabsorption of water in the ileum and cecum (Ikuno *et al*, 1995) following CPT-11 treatment. The intestinal data indicate that consumption of AAFA™ prior to and during CPT-11 treatment reduced intestinal damage, as reflected by less CPT-11 induced apoptosis in the crypts of the small intestine. The decrease in the number of apoptotic figures per crypt in CPT-11 treated mice fed 3% AAFA™ suggest that omega-3 polyunsaturated fatty acids have a role in protection of the intestine from genomic damage resulting in programmed cell death. This result provides evidence in support of a previous claim that supplementing the diet with omega-3 fatty acids protects against CPT-11 induced intestinal damage (Hardman *et al*, 1999a).

The CPT-11 induced enlargement of the livers of mice not fed AAFA™ could be due to damage to the liver resulting in inflammation, or to hypertrophy as a result of an increased demand to detoxify blood. Following CPT-11 treatment, humans exhibit increased serum glutamic oxalic transaminase levels (Rothenberg *et al*, 1996) and increased serum bilirubin (Murray and Kelly, 2001) indicating hepatotoxic damage. Drug induced toxic injury to the liver can produce inflammation (Damjanov and Linder, 1996). Thus, inflammation induced swelling is a likely cause for the increased liver weight. Consumption of 2% AAFA™ reduced CPT-11 induced liver inflammation as indicated by the correlation between a decreased level of the inflammatory eicosanoid, PGE_2_, in the liver and by the lack of liver hypertrophy.

The ability of AAFA™ consumed in the diet to significantly reduce liver PGE_2_ suggests that the production of inflammatory eicosanoids in the intestine may also be reduced. CPT-11 associated intestinal damage is mediated by excess eicosanoid production (Kase *et al*, 1997; Sakai *et al*, 1995, 1997). Thus, systemic suppression of eicosanoid production by consumption of omega-3 fatty acids may explain the reduced apoptosis in the duodenum seen in this study and the reduced intestinal damage reported in our previous study (Hardman *et al*, 1999b). More research is needed to establish the mechanism(s) involved.

Data from human studies indicate that neutropenia and/or anaemia occurred in 43% of patients treated with CPT-11 (Rothenberg *et al*, 1999). In mice that did not consume AAFA™, treatment with CPT-11 caused a significant reduction in the WBC count and RBC count. However, the data from this study indicate that consumption of 2% AAFA™ by mice prior to and during CPT-11 treatment resulted in RBC counts that were not significantly different from normal and in improved WBC counts.

Eighty-three per cent of humans also report asthenia (tiredness or weakness) as a side-effect of CPT-11 treatment (Rothenberg *et al*, 1999). In mice, asthenia could be demonstrated by behavioural changes such as lack of grooming. Thus, the ‘scruffy’ look of the mice that did not consume AAFA™ and that were treated with CPT-11 is likely due to this behavioural change. The ‘scruffy’ look was decreased in mice that consumed 1% AAFA™ and was completely eliminated in mice that consumed 2 or 3% AAFA™ suggesting that the mice treated with CPT-11 and fed AAFA™ felt better and continued normal grooming behaviour.

Micronuclei in the peripheral blood erythrocytes and apoptotic figures in the duodenal crypts were used as assays of genomic damage. Micronuclei are formed in blood cells following double-strand breaks in DNA resulting in chromosomal fragments left behind at mitosis (Vijayalaxmi *et al*, 1997). Apoptosis can result following unrepaired DNA strand breaks (Potten, 1992). CPT-11 binds to topoisomerase I and stabilises the topoisomerase I induced DNA strand breaks, leading to irreversible breaks in double-strand DNA in replicating cells (Slichenmeyer *et al*, 1993). The marked increase in micronuclei in peripheral blood erythrocytes and the increase in apoptotic cells in the duodenal crypts in mice treated with CPT-11 was therefore expected.

Omega-3 fatty acids have been shown to slow cell proliferation in normal tissues (Anti *et al*, 1994; Bartram *et al*, 1993; Calviello *et al*, 1999; Meydani *et al*, 1991) thus the observed reduction in the number of micronuclei in peripheral erythrocytes and in the number of apoptotic figures in the duodenal crypts of mice treated with CPT-11 and fed AAFA™ may simply be due to slower proliferation during the time of CPT-11 treatment. The fact that we observed less apoptosis in the crypt columns of the mice yet the number of cells in each crypt column was not changed also indicates that proliferation in the intestinal mucosa was slowed due to AAFA™ consumption.

The reduced number of erythrocytes with micronuclei in mice treated with CPT-11 and fed AAFA™ may also be associated with slowed proliferation due to a decreased demand for new erythrocytes. In this regard, Fisher and Black (1991) reported that the erythrocytes of mice fed 12% fish oil had higher levels of n-3 fatty acids and were less osmotically fragile than the erythrocytes of mice fed 12% corn oil. This could result in a longer lifespan for individual erythrocytes and a reduced need for new erythrocytes followed by a reduced need for proliferation of erythroblasts to maintain RBC counts in the peripheral blood. The marked dose dependent decrease in numbers of polychromatic (immature) erythrocytes in mice consuming AAFA™ and treated with CPT-11 (Figure 1, graph 10) supports the idea that fewer new erythrocytes were being produced to maintain the RBC counts in the peripheral blood of AAFA™ fed mice.

There was a dose response towards normal in mice that consumed 1 or 2% dietary AAFA™ for: apoptotic figures in the duodenal crypts, liver weight, WBC and RBC counts. The variance from the dose response for 3% dietary AAFA™ for liver weight and for WBC counts may indicate that addition of more than 2% AAFA™ does not increase the effectiveness of the diet supplement for these parameters. Two per cent AAFA™ in the diet of the mice is equal to 18 calories AAFA™ per 430 calories of the diet or 4.1% of the calories in the diet from AAFA™. In human terms, consumption of 9 g of AAFA™ per day in an 1800 calorie diet would be 81 out of 1800 or 4.5% of the calories from AAFA™, 8 g per day would equate to 72 out of 1800 or 4.0% of the calories from AAFA™. Thus consumption of 8 to 9 g of AAFA™ per day by humans would equate to the 2% AAFA™ diet fed the mice in this study. Anti *et al* (1992) have reported that humans can safely consume 10 g fish oil per day without ill effects while Burns *et al* (1999) report administration of up to 21 g per day of an omega-3 fatty acid product. The product used in the Burns trial contained 626 mg EPA + DHA per gram thus the 21 g per day dose equaled 13.1 g EPA + DHA per day. Based on the Burns phase I study, it seems that 8 or 9 g per day of AAFA™ is a feasible dose for humans.

Clinical trials must be conducted to determine if the beneficial effects of omega-3 fatty acid supplements seen in mice can be translated to humans undergoing cancer therapy. Clinical trials using a daily supplement of 8 g per day of AAFA™ for cancer patients undergoing chemotherapy seem warranted based on the preclinical studies described herein and these clinical trials are being initiated.
